# Typical Lateral Interactions, but Increased Contrast Sensitivity, in Migraine-With-Aura

**DOI:** 10.3390/vision2010007

**Published:** 2018-02-09

**Authors:** Jordi M. Asher, Louise O’Hare, Vincenzo Romei, Paul B. Hibbard

**Affiliations:** 1 Department of Psychology, University of Essex, Wivenhoe Park, Colchester CO4 3SQ, UK; 2 Faculty of Health and Social Sciences, Lincoln University, Brayford Way, Brayford Pool, Lincoln LN6 7TS, UK; 3 Dipartimento di Psicologia and Centro studi e Ricerche in Neuroscienze Cognitive, Università di Bologna, Campus di Cesena, 47521 Cesena, Italy

**Keywords:** migraine, lateral inhibition, collinear facilitation, contrast sensitivity

## Abstract

Individuals with migraine show differences in visual perception compared to control groups. It has been suggested that differences in lateral interactions between neurons might account for some of these differences. This study seeks to further establish the strength and spatial extent of excitatory and inhibitory interactions in migraine-with-aura using a classic lateral masking task. Observers indicated which of two intervals contained a centrally presented, vertical Gabor target of varying contrast. In separate blocks of trials, the target was presented alone or was flanked by two additional collinear, high contrast Gabors. Flanker distances varied between 1 and 12 wavelengths of the Gabor stimuli. Overall, contrast thresholds for the migraine group were lower than those in the control group. There was no difference in the degree of lateral interaction in the migraine group. These results are consistent with the previous work showing enhanced contrast sensitivity in migraine-with-aura for small, rapidly presented targets, and they suggest that impaired performance in global perceptual tasks in migraine may be attributed to difficulties in segmenting relevant from irrelevant features, rather than altered local mechanisms.

## 1. Introduction

Migraine is a debilitating neurological disorder affecting approximately 14% of the population [1,2], and attacks are often characterised by an aversion to light and sound [3]. Some individuals with migraine experience sensory disturbances preceding their attack, known as migraine aura, which are most commonly experienced in the visual modality [4,5]. While the pathophysiology of migraine is still not fully understood, these differences in visual processing suggest that the visual pathways may play a key role. Consequently, perceptual tasks are often used to explore the behavioural differences in brain functioning [6,7]. Sensory processing differences between migraine and control groups have been found for a multitude of psychophysical tasks, including sensitivity to temporal and spatial contrast [8,9], colour and orientation [5,10], global motion and form coherence thresholds [11,12,13] and adaptation to prolonged stimulation [7,14,15]. These effects on sensory processing are not restricted to the time of the attack itself, and differences in performance are also shown interictally (in between attacks) [16,17].

Migraine has been associated with a state of cortical hyper-responsiveness, the idea that the brain responds more strongly to sensory stimuli in migraine [18,19,20]. Explanatory hypotheses to account for the neurophysiological abnormalities found in migraine sufferers include reduced inhibitory control between neurons [10] and an inability to ignore internal noise [16,21] or as a result of preactivation from the brainstem and/or thalamus [22]. Many aspects of low-level visual processing rely on excitatory and inhibitory lateral interactions between neurons, for example, visual search, border segmentation [23], and the integration and segmentation of local features into globally coherent forms [24]. The current study assesses the extent to which these lateral interactions, both excitatory and inhibitory, might differ in their strength and spatial extent in migraine-with-aura.

### 1.1. Altered Lateral Interactions in Migraine

Physiological evidence supports the idea of impaired lateral interactions in migraine. Bridge et al. [25] found reduced levels of the inhibitory neurotransmitter gamma-aminobutyric acid (GABA) in a group with migraine-with-aura and visual sensitivity and argued that the reduced GABA levels disrupt the coupling between excitatory and inhibitory activity in visual areas. Consistent with these reduced GABA levels, there is also psychophysical evidence that lateral interactions between neural responses may be different in migraine, although the pattern of results across behavioural tasks is inconsistent [16]. Coppola et al. [22] recorded visual evoked potentials (VEPs) using electroencephalogram (EEG) electrodes placed on the back of the head, while observers viewed radial windmill dartboard or partial windmill visual patterns. The study found that for migraine observers, VEP amplitudes were reduced during the intericatal period compared to controls but were normalised during the ictal period (during an attack). This provides evidence for an imbalance between excitation and inhibition for individuals with migraine during the interictal period [22]. Furthermore, sensitivity to orientation differences is reduced in migraine [26]. This task depends on lateral inhibition as a mechanism for sharpening orientation tuning [27], and orientation sensitivity is known to correlate positively with GABA concentration in the visual cortex [28]. Other studies have reported no difference in inhibitory interactions in migraine [18]. Using contrast detection against a high-contrast background to assess the effects of contrast gain control, Battista et al. [6] compared inhibitory interactions between migraine and control groups using the Chubb illusion [29]. This task involves judging the contrast of a central texture of varying contrast within an annular surround. When the surround is higher in contrast, the centre appears reduced in contrast. Surround suppression relies on lateral interactions between neurons [29]. Increased surround suppression was found for moving stimuli in the migraine groups compared to the controls, but no difference was found for static targets.

### 1.2. Masking in Migraine

Visual masking involves introducing noise to a stimulus so as to make the target harder to detect. Masks can be presented before (forward masking) or after (backward masking) the target. If spatial inhibitory interactions in migraine groups are reduced, then the degree of masking would also be expected to be reduced [30]. However, some evidence indicates that backward masking is no different in migraine groups in comparison with control groups [10,31]. Wagner et al. [32] found increased masking in migraine-with-aura and attributed this to increased levels of multiplicative noise.

### 1.3. Global Processing in Migraine

Migraine groups also show a deficit in global processing tasks such as global form [7] and global motion detection [21]. Global form perception relies on the effects of an association field, a local network of inhibitory and excitatory interactions that group together individual elements in order to detect extended contours [24]. The association field is important in allowing for the detection of globally defined shape [24] at higher stages of visual processing. The impoverished perception of global shape and form in migraine are consistent with an altered pattern of inhibitory and/or excitatory lateral interactions.

### 1.4. Current Study

Physiological and psychophysical evidence suggest that lateral interactions may differ in migraine, and these differences are likely to be related to the reduced ability to detect global form. The aim of the current study was to use a detailed psychophysical approach to directly assess the strength and spatial extent of these interactions in migraine-with-aura. We used the technique pioneered by Polat and Sagi [33] to do this. Observers were required to detect a central Gabor at varying levels of contrast, presented either alone or in the presence of high-contrast, collinear flankers of the same orientation and spatial frequency as, and spaced between 2 and 10 wavelengths from, the central target. Polat and Sagi found reduced contrast sensitivity when the flankers were very close to the target, consistent with visual masking effects, but improved sensitivity for flankers at a distance of up to 10 wavelengths, indicating the presence of local excitatory interactions. This design will therefore allow us to determine whether inhibitory and excitatory interactions differ in their magnitude or spatial extent in migraine-with-aura.

## 2. Materials and Methods

### 2.1. Participants

A total of 76 observers were tested. The categorisation of observers into groups was undertaken using the criteria of the Headache Classification Subcommittee of the International Headache Society [3]. All observers completed the experiment regardless of group. However, because differences in masking have been found to be greater in migraine-with-aura than in those without aura [32,34], only the with-aura group was investigated. Inclusion as a control observer required no symptoms of severe headache, migraine or aura. Migraine observers were tested interictally and were required to be free from migraine for 3 days either side of the day of testing. No observers used prophylactic medication for migraine. The data for two migraine observers were excluded as a result of experiencing an attack within 3 days of their testing day. The duration of testing was for 50 min on one day only. After the classification process, there were 31 controls (19 females, mean age of 22.8) and 24 with migraine-with-aura (22 females, mean age of 26.6; see Table 1); 18 observers were excluded after being assessed as either migraine without aura, non-headache-free controls or migraine-with-aura not meeting inclusion criteria. All experiments were conducted in accordance with the World Medical Association Declaration of Helsinki (2013) and were approved by the University of Essex ethics committee (Application No. VR1403). All observers gave written, informed consent and received payment for their participation.

### 2.2. Apparatus

Stimuli were presented using on a Sony Trinitron 2100 monitor with a screen resolution of 1280 × 1024 pixels and a vertical refresh rate of 100 Hz. One pixel subtended 0.7 arc min. A Datapixx CRT Driver (Vpixx Technologies, Saint-Bruno, QC, Canada) was used to achieve 16-bit control of contrast levels. Stimuli were generated and presented using MATLAB and the Psychophysics Toolbox extensions [35,36,37]. Responses were made via the left and right arrow keys on a standard keyboard.

### 2.3. Stimuli

**Figure 1:**
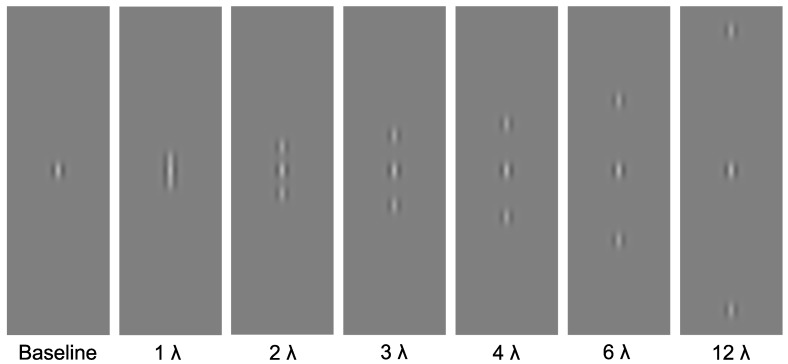
Experimental stimuli. Central Gabor target presented alone, and in the presence of high-contrast, high-spatial-frequency flanking Gabors at one of six distances (1λ, 2λ, 3λ, 4λ, 5λ, 6λ or 12λ).

Stimuli were presented on a mid-grey background. The target stimuli were centrally presented, vertically oriented Gabor patches, with a spatial frequency of 4 cycles per degree. The contrast of the target was manipulated: there were seven contrast levels (0.0075%, 0.015%, 0.0225%, 0.03%, 0.045%, 0.06% and 0.09%; Michelson Contrast). The flankers consisted of two collinear high-contrast, high-spatial-frequency Gabors with a spatial frequency of 4 cycles per degree. Flankers were positioned above and below the target at one of six distances (1, 2, 3, 4, 6 or 12 wavelengths) from the target. A baseline condition was also included, in which the target was presented alone (without flankers). See Figure 1 for example stimuli.

### 2.4. Procedure

**Figure 2:**
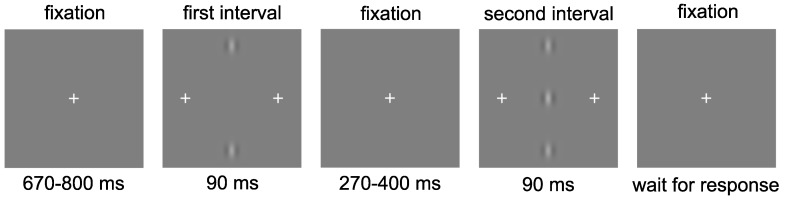
Observers were required to select which of two presentations contained the central Gabor target. In this example, the target is presented in the second interval.

Observers were positioned at a viewing distance of 1.5 m from the display. The task consisted of a two-alternative-forced-choice (2AFC) procedure to identify which of two intervals contained the target. The flankers (if present) appeared in both intervals (Figure 2). Stimulus presentation began with a central fixation cross displayed for a duration of between 670 and 800 ms, randomly selected for each trial. The first interval was presented for 90 ms, followed by a central fixation cross for a randomly selected duration of between 270 and 400 ms, followed by the second interval also presented for 90 ms. During the stimulus presentation, additional crosses were presented either side of the central target to alert the observer that a presentation was in progress and to aid with identifying where the target would appear. These crosses were located at 70 arc min to the left and right of the central target location. There were 20 repetitions of each contrast level, resulting in a total of 140 trials for each flanker distance. Each distance was presented in a separate block, resulting in seven blocks, one for each of the six flanker distances and one for the baseline (no flanker) condition. Contrast levels were randomised within each block. The block order was also randomised for each observer.

## 3. Results

The responses from each observer were converted into the probability of being correct out of the total number of trials for each condition. A generalised linear mixed effects model was used to analyse the data, using the *fitglme* function in MATLAB. We first compared the contrast sensitivity for the Gabor target in the absence of flankers, using a model with contrast as a fixed covariate, group as a categorical factor, and observer as a random factor, with random intercepts and contrast slopes. A probit link function was used to convert the dichotomous responses into a continuous variable. This technique considers between-individual variation as a random factor and has the advantage of higher statistical power compared to traditional analysis using a two-level approach [38].

**Figure 3:**
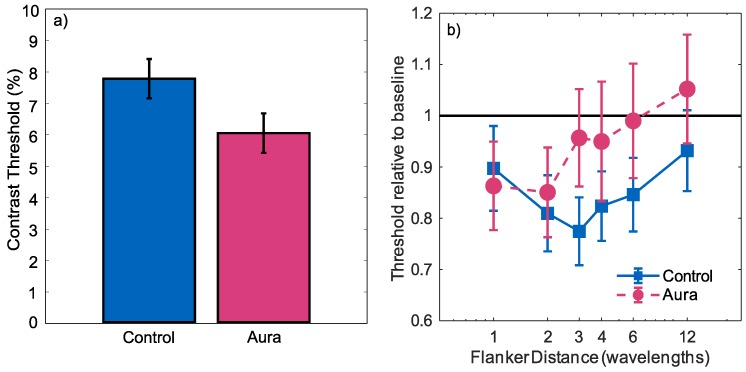
(**a**) Contrast detection thresholds (75% correct) for aura and control groups for Gabor targets in the absence of flankers; (**b**) contrast detection thresholds (75% correct) for stimuli with flankers present. Thresholds are plotted relative to the condition’s baseline (marked by the horizontal black line). Normalisation was undertaken independently for the control and migraine groups. Error bars indicate ±1 standard deviation.

There was a significant effect of contrast (β=29.141;t(402)=9.076;p<0.001), indicating that the number of correct responses tended to increase with increasing contrast. There was no significant effect of group (β=−0.391;t(402)=−1.274,p=0.20), but there was a significant group by contrast interaction (β=12.313;t(402)=2.102;p=0.0036). Performance increased more quickly with contrast for the migraine-with-aura group, indicating greater contrast sensitivity. This difference was summarised by calculating a 75% threshold from the estimated intercept and slope for each group. Confidence limits were then calculated using the bootstrap method proposed by [38]. For each of 1000 bootstrap samples, the estimated slope and intercept as well covariance of these parameters were used to create the intercept and slope estimates for a population of simulated observers of the same size as each group. For each simulated observer, the probit linking function was used to specify the expected proportion of correct responses at each contrast, and a simulated psychometric function was generated by random sampling of a binomial distribution. A group 75% threshold was then calculated using the same generalised linear mixed effects model as was used for the true data. This process was used to create 1000 simulated thresholds, from which the standard deviation of the threshold estimates could be calculated. The thresholds are plotted in Figure 3a, showing the increased contrast sensitivity for the migraine-with-aura group in comparison with the control group (*p*<0.001).

Relative 75% thresholds for stimuli with flankers are plotted as a function of the flanker distance in Figure 3b. Thresholds were normalised independently for each condition by dividing each threshold by that estimated in the absence of flankers. These results therefore show the change in thresholds induced by the flankers, with values greater than 1 indicating inhibition and values less than 1 indicating facilitation. For both groups, the presence of flankers tended to reduce thresholds, with the degree of facilitation decreasing as the distance of the flankers from the target increased. This facilitation was analysed using a generalised linear mixed effects model with a probit linking function. Contrast, flanker distance and participant group were used as predictors. Three interaction terms were also included in the model. Contrast-by-flanker distance was included to test whether the slope of the psychometric function was affected by the flanker distance. Group-by-flanker distance was included to determine whether the flanker effect differed between the two groups. Finally, group-by-flanker distance-by-contrast was included to determine whether any effects of the presence of flankers on the shape of the psychometric function differed between the two groups.

The performance increased with increasing contrast (β = 31.46; *t*(2428) = 9.912; *p* < 0.001), and decreased with increasing flanker distance (β = −0.05807; *t*(2428) = −3.680; *p* < 0.001). There was no main effect of group (β=−0.02525;t(2428)=−0.1716;p=0.8638), and there were no significant distance-by-contrast (β=0.2389;t(2428)=0.7820;p=0.434), group-by-flanker distance (β = 0.02017; *t*(2428) = 0.8400; *p* = 0.4010) or group-by-flanker distance-by-contrast (β = −0.4107; *t*(2428) = −1.082; *p* = 0.279) effects. These results show a facilitatory effect of the flankers, that this decreased with increasing distance, and that the degree of facilitation did not differ between the two groups.

## 4. Discussion

The purpose of this study was to directly investigate the lateral interactions in individuals with migraine with aura, to determine whether the deficits experienced were consistent with a reduced inhibitory effect. We found no evidence of differences in inhibitory or excitatory lateral interactions in those with migraine compared to the control group, consistent with previous findings [31]. This does not support the hypothesis that cortical hyperexcitation is due to a lack of inhibition between units. The advantage of the current study is that this has been demonstrated for band-limited stimuli of the same orientation, controlling for potential confounding visual processes [33,39]. Aurora and Wilkinson [20] suggested that collinear facilitation might be abnormally strong in migraine. While there was no evidence to support this hypothesis in the current study, our results did show that individuals with migraine-with-aura exhibited increased performance on the baseline contrast detection task.

### 4.1. Contrast Sensitivity

One of the most basic ways in which visual performance might differ interictally between migraine and control groups is in contrast sensitivity. While there is some evidence that contrast sensitivity is reduced in individuals with migraine [40,41,42,43], the findings are inconsistent [16]. Some studies have found evidence for poorer contrast sensitivity in migraine when testing with large [44] or peripherally presented stimuli, but not when testing with centrally presented stimuli [9]. There is also evidence for reduced performance in migraine compared to controls for low-spatial-frequency stimuli but not high-spatial-frequency stimuli [8]. However, studies using small, briefly presented stimuli, similar to those used here, have found contrast sensitivity in migraine to be as good as controls in [34] or better than controls in [5]. This discrepancy may be related to the variation in sensitivity found across the visual field in migraine [45] and the variety of psychophysical techniques used across studies [16]. Increased contrast sensitivity, as found in the current study, is consistent with the idea of hyperexcitation in migraine [10]. This is inconsistent with other studies demonstrating reduced-contrast sensitivity in those with migraine, using the Cambridge Low Contrast Gratings (e.g., [5,46]). It has been suggested that the reduced contrast sensitivity in migraine shown in some studies can be accounted for by increased internal noise levels in the visual system [34]. The Cambridge Low Contrast Gratings are a much larger stimulus than the Gabor patches used in the current study, and as there is variation across the visual field in migraine [45], this might have some bearing on the differences between previous research and the current findings.

### 4.2. Inhibition and Lateral Interactions

Many aspects of low-level visual processing rely on excitatory and inhibitory lateral interactions between neurons, for example, visual search, border segmentation, and defining contours [23]. Inhibition has been proposed to mediate the strength of sensory activation within the primary visual cortex (V1). Because populations of neurons are sensitive to different ranges of spatial frequencies, when the frequency of the incoming sensory information matches a channel’s frequency tuning, these channels respond. Channels whose frequency tuning does not correspond to the incoming sensory information do not respond. In addition to these direct excitatory responses to external stimulation, the channels also receive signals from neighbouring neural populations that code for similar spatial frequencies or orientations [33]. These interactions may be different in migraine, for whom migraine is proposed to account for the deficit in performance in some visual detection tasks [16]. However, the behavioural evidence is inconsistent; for example, no difference in inhibitory interactions in migraine was found for a contrast masking task, in which observers were required to detect a rapidly presented target on a high-contrast mask [18].

Furthermore, while Battista et al. [6] found differences in inhibitory interactions between migraine and control groups using the Chubb illusion [29], this was only the case for moving stimuli, and increased, rather than reduced, inhibition was found. Both groups performed at the same level when static stimuli were presented. McKendrick et al. [17] showed increased surround suppression in migraine using a drifting version of the Chubb illusion, consistent with a reduced level of short-range inhibition measured interictically using visually evoked potentials [22]. In addition, there were effects of the migraine cycle—specifically less surround suppression in the migraine group 2 days before the onset of the attack. However, the reduced performance for moving stimuli may not be completely accounted for by reduced lateral inhibition. For instance, evidence suggests that motion after-effects are enhanced in migraine [14]. The motion after-effect is a visual illusion of motion observed after viewing moving stimuli for an extended period. After adapting to motion in one direction, observers perceive motion in the opposite direction whilst viewing a stationary stimulus [47]. It has been argued that increased motion after-effects in migraine can best be explained by reduced excitatory intracortical interactions [14,15,46]. If this is the case, we might have predicted a decreased flanker facilitation effect in migraine-with-aura ; however, this was not found in the current study. While no significant difference was found, the curve appeared shifted leftwards for the migraine aura group relative to the control group (Figure 3b). This shift would not represent a change in the strength of the facilitation effect but in the spatial area over which it operates. In this case, a significant shift would have represented a reduction in the spatial extent of facilitatory connections.

In contrast, the physiological evidence for reduced levels of the inhibitory neurotransmitter in individuals with migraine-with-aura is robust [25]. Because reduced GABA is argued to disrupt the excitatory and inhibitory activity in the early visual areas [25], it has been predicted that individuals with migraine should perform worse in tasks, such as orientation discrimination, that require strong local inhibitory interactions. Sharpening of the orientation tuning for individual receptive cells depends on lateral inhibition [27], and orientation sensitivity is known to correlate with GABA concentration in the visual cortex [28]. Tibber et al. [26] tested this and found as predicted that sensitivity to orientation differences was reduced in migraine. However, the orientation deficit was only found for oblique, not cardinal, orientations; therefore the authors ascribed this finding to difficulties in pooling inhibitory/excitatory interactions, rather than impaired lateral inhibition alone. Additionally, Wilkinson and Crotogino [48] showed a trend for poorer orientation selectivity in those with migraine; however, as this was not statistically significant, the authors emphasise that this is inconclusive evidence for reduced lateral inhibition in migraine.

In this study, we predicted that should individuals with migraine experience a difference in the balance between inhibitory and excitatory interactions in V1, then the pattern of inhibition and facilitation in this lateral masking task would differ from that of controls. Our results found no difference between migraine and control groups. Our study adds to the literature that suggests lateral inhibitory processes may not be exclusively responsible for the perceptual differences experienced in those with migraine. While reduced GABA levels are argued to cause disruption in excitatory and inhibitory activity within visual areas and are correlated with cortical excitability [25], this does not appear to impact lateral interactions in behavioural tasks in a consistent and reliable manner. Wagner et al. [34] propose that reduced cortical suppression may be reduced through GABAergic inputs, and this may also impact the efficiency with which individuals with migraine can exclude noise.

### 4.3. Global Processes and External Noise

Given the lack of any differences in lateral interactions from our study, the alternative explanation for impaired performance in migraine is the failure to ignore external noise when performing global integration tasks [21,32,34]. Global processes have been studied in migraine, using both global form [7,12] and global motion tasks [21,42]. Global form perception in migraine has been assessed with Glass patterns [7,12]. Glass patterns typically consist of pairs of elements aligned to show a global form. Noise was introduced to these stimuli by varying the orientation of the elements relative to each other. These studies demonstrated higher thresholds (poorer performance) in migraine groups for Glass pattern detection. This result might be taken as evidence that those with migraine are poorer at contour integration, a process that depends on the lateral interactions in the association field (e.g., [24,49]). However, no evidence for differences in lateral interactions was found in the current study. The difference between the Glass patterns and the current study stimulus is the inclusion of additional noise elements in the former. In the Glass pattern task, the aim is to detect the coherent orientation of the pattern, with noise typically added by increasing the proportion of randomly oriented “noise” elements. This involves at least two processes, the contour integration, and also the discrimination of the elements of coherent orientation against the noise elements.

Analogously, motion coherence is typically studied using random-dot kinematograms. In a typical stimulus, there are a proportion of signal dots that move in one direction while the remainder move randomly. Difficulty is manipulated by varying the number of signal dots; as it is not possible to identify the motion from a single dot, the mean direction is inferred by segregating out the noise dots and integrating the coherently moving signal dots [21]. People with migraine have shown a deficit in judging the direction of motion in these kinds of tasks [11,12,21,42]. In these tasks, local motion needs to be detected, the coherent direction integrated, and the randomly moving “noise” elements segregated from the coherently moving stimuli [50]. Motion coherence can also be studied using a paradigm of equivalent noise. In this method, rather than assigning signal and noise dots, all dots contribute equally to the signal, and the difficulty of the task is increased by varying the distribution of the direction of motion of the dots (see [21]). Observers with migraine were found to perform normally on equivalent noise motion coherence tasks. This suggests that rather than a deficit in pooling signals, people with migraine may have difficulty segmenting signals from randomly moving “noise” elements in the more typical global motion task. This suggests that deficits in migraine may not be due to lateral interactions needed to integrate the coherent motion but are from the exclusion of noise from the signal. The inability to exclude noise from signals has been suggested as the reason for the differences seen in migraine in masking studies [34].

There are also temporal factors that affect differences in lateral interactions in migraine studies, typically those finding large robust differences between migraine and control groups are those involving motion, such as drifting gratings in the Chubb illusion [6,17], prolonged motion after-effects [14,15,46], and poorer performance in tasks involving the discrimination of motion against noise backgrounds [21,42]. Increased masking in migraine is found at all stimulus onset asynchronies, but the exact timing of the target relative to the mask makes a difference—the masking effect is greatest for 60–100 ms intervals [32]. The authors modelled this effectively by increasing both the variable representing gain control instead of the post-gain-control noise variable in their perceptual template model. The post-gain-control noise variable relates to the shape-encoding mechanism in the model. GABA is related to the gain control of the visual system, and although GABA is associated with orientation specificity, it might not be directly relevant to this higher-level shape mechanism [51].

## 5. Conclusions

Contrast sensitivity was increased in those with migraine with aura, which was consistent with cortical hyperresponsivity. This is contrary to other measures of contrast sensitivity in migraine. The difference between the current study and previous findings could be related to the stimulus size, as performance varied across the visual field in migraine, and orientation-specific deficits tended to be for lower-spatial-frequency stimuli. This study does not support the hypothesis of altered lateral interactions in those with migraine-with-aura. It is suggested that the overall patterns of poorer performance in those with migraine in global tasks could be due to a reduced ability to exclude noise, rather than differences in lateral interactions per se.

## Figures and Tables

**Table 1:** Migraine observers’ reports of clinical features.

Observer	Sex	Age	Age Started	Prior Attack	Frequency per Month
OB8	F	23	16	1 month	1–3
OB12	F	27	10	2 weeks	1–3
OB14	F	60	19	3 months	<1
OB16	F	52	14	5 days	1–3
OB17	F	22	17	5 days	1–3
OB20	M	30	12	4 months	5 or more
OB21	F	41	24	9 days	5 or more
OB22	F	21	10	4 days	<1
OB24	F	36	23	7 days	1–3
OB25	F	29	3	20 days	1–3
OB28	F	30	13	3 months	<1
OB29	F	55	40	6 days	1–3
OB30	F	25	9	8 days	1–3
OB31	F	23	19	1 month	1–3
OB33	M	20	12	7 days	1–3
OB48	F	18	NDI	1 month	5 or more
OB50	F	21	NDI	7 days	<1
OB63	F	18	13	1 month	1–3
OB64	F	19	17	7 days	5 or more
OB66	F	19	16	A few months	1–3
OB71	F	20	11	> 3 days	<1
OB73	F	18	15	6 days	5 or more
OB75	F	19	16	3 months	<1
OB76	F	18	NDI	> 3 days	NDI
Excluded	F	19	16	1 day	1–3
Excluded	F	18	14	2 days	1–3

NDI—non-disclosed information.

## Abbreviations

The following abbreviations are used in this manuscript:

GABA	Gamma-aminobutyric acid
2AFC	Two-alternative-forced-choice
V1	Primary visual cortex
VEP	Visual evoked potential
EEG	Electroencephalography
